# Studying the mechanistic links between microtubule disruption and tauopathy protein pathogenesis

**DOI:** 10.1002/alz.084089

**Published:** 2025-01-03

**Authors:** Wen Zeng, Zhuohao He

**Affiliations:** ^1^ Shanghai Institute of Organic Chemistry, Chinese Academy of Sciences, Shanghai China; ^2^ Shanghai Institute of Organic Chemistry, Chinese Academy of Sciences, Shanghai, Shanghai China

## Abstract

**Background:**

Pathological tau plays critical roles in many neurodegenerative diseases (NDD), including Alzheimer’s disease (AD). However, the mechanisms underlying the initial tau pathogenesis are largely unknown. Extensive tau pathology has been observed in the brains with chronic traumatic encephalopathy (CTE), suggesting repeated traumatic brain injury (rTBI) correlates with tau pathogenesis. However, the mechanistic links are missing.

**Method:**

We developed a rTBI mouse model, combined with our recently developed seeding models, to investigate whether and how rTBI is correlate with initial tau pathogenesis. Moreover, we utilized cryo‐electron microscopy (Cryo‐EM) and chemistry synthesis approaches to examine the co‐factors that are involved in tau fibrilization.

**Result:**

We found rTBI induced tau pathogenesis and spreading, and the microtubule disruption is involved in such process. Pharmacologically disrupting microtubule induced tau pathogenesis, which could be rescued by microtubule stabilizer. Further study showed microtubule disruption induced Golgi apparatus (GA) fragmentation, which would be also observed in tauopathy brain tissues. Moreover, GA contents could directly induce the tau fibrilization. When inoculated into WT mouse brains, these fibrils transformed endogenous physiological tau into pathological status, mirroring the tau extracted from human patient brains. Subsequent Cryo‐EM structures showed the tau fibrils recapitulated certain pathological tau harbored similar folds as the tau fibrils extracted from human AD and chronic traumatic encephalopathy (CTE) brains.

**Conclusion:**

Our studies suggest microtubule disruption induces GA fragmentation, resulting in the leakage of GA contents, that could trigger tau fibrilization and pathogenesis. Our results suggest keeping microtubule and GA integrity as novel intervention strategies for the treatment of tauopathy diseases.

